# Low concentration of BPA induces mice spermatocytes apoptosis via GPR30

**DOI:** 10.18632/oncotarget.16923

**Published:** 2017-04-07

**Authors:** Chaoliang Wang, Jianxiang Zhang, Qi Li, Tianbiao Zhang, Zishi Deng, Jing Lian, Donghui Jia, Rui Li, Tao Zheng, Xiaoju Ding, Fan Yang, Chao Ma, Rui Wang, Weixing Zhang, Jian Guo Wen

**Affiliations:** ^1^ Department of Urology, The First Affiliated Hospital of Zhengzhou University, Zhengzhou, Henan Province, China; ^2^ Department of Breast Surgery, The First Affiliated Hospital of Zhengzhou University, Zhengzhou, Henan Province, China; ^3^ No.1 Middle School of Yiyang, Yiyang, Hunan Province, China

**Keywords:** BPA, GPR30, Erk1/2, EGFR-MAPK pathway, spermatocyte apoptosis

## Abstract

Bisphenol A (BPA) acts as xenoestrogen and has a great impact on disorders of human reproductive system. However, the mechanism through which BPA can affect human testicular function remains to be identified. GPR30 is a novel membrane estrogen receptor with high-affinity and low-capacity binding to estrogens. We demonstrated that estrogen receptor α (ERα), estrogen receptor β (ERβ) as well as GPR30 are expressed in mouse spermatocyte-derived GC-2 cells using Real-time PCR. We treated the cells with different doses of BPA and found that even low doses of BPA can inhibit GC-2 cell growth using MTT assay. To make sure which receptor is responsible for the biological function of BPA, we used ER down-regulator ICI and indicated that BPA could bind to GPR30. We also observed that BPA was able to induce Erk1/2 phosphorylation in GC-2 cells and proved that this process was mediated by GPR30–related EGFR-MAPK pathway using western blot. By Real-time PCR, we found that the expression of *c-Fos* was up-regulated and *Cyclin D1* gene was down-regulated, in the presence of BPA and ICI. The results of MTT assay, comet assay and flow cytometry indicated that the activation of GPR30 induced by BPA inhibited the cell growth and induced cell apoptosis and ICI, GPR30 siRNA, EGFR inhibitor (AG), and MAPK (PD) inhibitor could partially reverse this effect. Immunohistochemistry on the testis of BPA –damaged mice showed that BPA induced spermatocyte apoptosis without affecting the seminiferous tubules and spermatocyte. In conclusion, BPA triggered spermatocyte apoptosis via GPR30.

## INTRODUCTION

It has been demonstrated that many man-made chemicals presented in food and environment are hormone-like pollutants, which can disrupt the endocrine systems of animals and hence are called environmental endocrine disrupting chemicals (EDCs) [1–3]. Most of the EDCs act as xenoestrogen and exhibit the abilities to mimic, antagonize, or alter the action of endogenous estrogen, and then jeopardize the reproductive capacities of various animals [4–6]. Bisphenol A (BPA), the major xenoestrogen generated by human activities, was broadly distributed in environment.

BPA is a high production volume chemical used in the manufacture of polycarbonate plastics and epoxy resins, which can be used in baby and water bottles, food container linings, beverage cans, medical tubing, dental fillings and other applications [7, 8]. Once it has contaminated the environment, BPA is expected to be more persistent in water and soil [9]. Dietary ingestion is considered the primary source of general population exposure to BPA. Other exposure sources may include water, air, and dust [10–13]. As a result, BPA exposure is widespread in the general populations.

BPA has been shown to alter endocrine function through multiple pathways [15]. In experimental animal studies, BPA treatment has been shown to accelerate growth and puberty, to disrupt embryonic development [16], and to induce aneuploidy [17]. BPA levels were positively correlated to the levels of testosterone and androstenedione, suggesting an interaction between androgen and the metabolism of BPA [18]. BPA has also been reported *in vitro* and *in vivo* studies to affect the male reproductive system including testes, epididymis, seminal vesicles, and prostate gland [19–23]. These lines of evidences strongly suggested that BPA can harm human reproductive health by acting as an endocrine disruptor.

Many studies have indicated that estrogens have a role in the regulation of testicular function. The absence of estrogen receptors (ERs) causes adverse effects on spermatogenesis and steroid genesis [24–26]. Xenoestrogens can mimic or antagonize the activity of physiological estrogens and have also been shown to affect testicular gene expression [24–27]. The suggested mechanism of xenoestrogen is thought to exert their estrogenic effects primarily by binding to the ER [28–30], which is belong to the nuclear receptor superfamily [31–33]. The mechanism by which BPA exerts its biological actions has been proposed. BPA should mimic or compete with endogenous estrogens, binds to both estrogen receptors (ERs) α and β (ERα and ERβ), which have been reported as the foremost receptors [8, 15, 34–36]. So, the research has mainly focused on the ability of BPA to affect specific cells through binding these nuclear receptors, although the binding affinity of BPA to estrogen receptor-α (ERα) or ERβ is 10,000-and 1,000-fold lower than that of estradiol (E2), respectively [37].

Recently, a large amount of evidence has demonstrated that estrogens not only can function through the classic genomic mechanism mediated by ERs but also can trigger rapid responses that involve transduction pathways through the non-genomic mechanism [38]. Some researches found that the G protein-coupled receptor-30 (GPR-30), a seven-transmembrane receptor structurally unrelated to the nuclear ERs, mediates rapid actions of estrogens [39–43]. The discovery of GPR30 has generated a great deal of interest to toward the identification of unknown functions and mechanisms triggered by estrogen outside the nucleus. GPR30 is a possible candidate for rapid estrogen signaling based on the observations that it mediates Erk activation and c-fos expression in an ER-independent manner [42, 44]. Some evidence suggests that BPA also binds to GPR30 and mediates Erk activation [45, 46]. However, the mechanisms by which BPA can bind to GPR30 and influence male fertility and spermatogenesis remain uncertain. Therefore, it is reasonable to hypothesize that BPA binds GPR30 to mediate non-genomic estrogenic actions and thus to alter these rapid signals. The aims of the present study are to investigate the biological function and signaling pathway of GPR30 influenced by BPA in mice spermatocyte.

## RESULTS

### The expression of estrogen receptors in GC-2 cell lines

**Figure 1:**
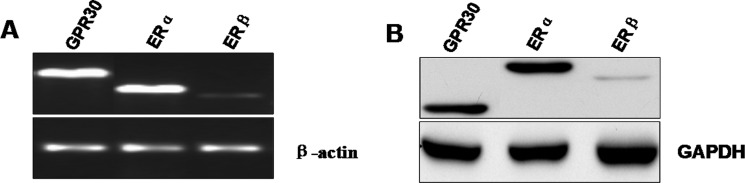
Expression of estrogen receptors at mRNA and protein levels in the mouse GC-2 cells (**A**) ERα, ERβ and GPR30 mRNA expression in GC-2 cells was analyzed by real-time PCR. The PCR products were resolved on 1% agarose gel electrophoresis and visualized by ethidium bromide staining. β-actin was used as control gene. (**B**) Western blot analysis of ERs was performed on 30 μg of total proteins extracted from GC-2 cells. Specific antibody for ERα, ERβ and GPR30 are representative of three independent experiments with similar results. GAPDH was used as a loading control.

To define ERs expression in mouse spermatocyte derived cell line, we analysed the relative mRNA expression levels of ERα, ERβ and GPR30 in cultured GC-2 cell lines using real-time PCR. The results demonstrated that GC-2 cells express both ERs isoforms as well as GPR30, while the level of ERβ isoforms was weaker compared to that of ERα or GPR30 (Figure 1A). We also confirmed the result by Western blot analysis, using specific antibodies against the ERα, ERβ and GPR30 isoforms (Figure 1B).

### Low dose of BPA induced inhibition of GC-2 cell growth

**Figure 2:**
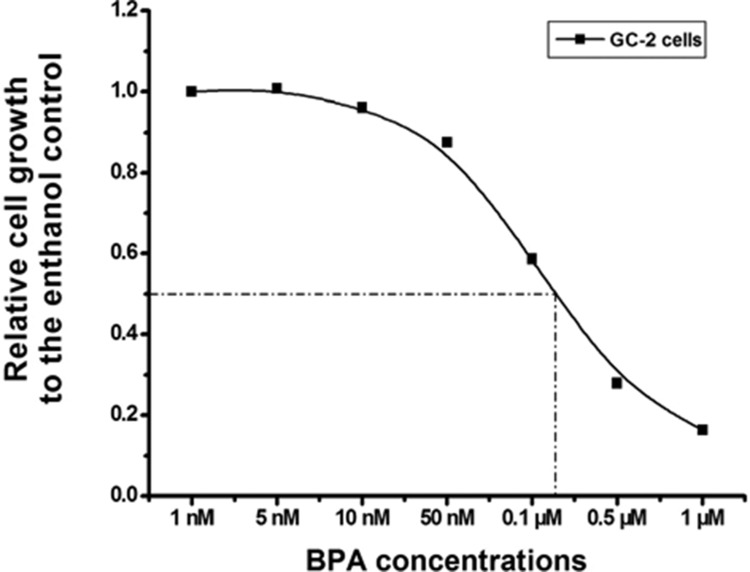
Dose-dependent inhibition of GC-2 cells growth induced by BPA GC-2 cells planted in 96-well plate were treated with 1 nM-1 μM BPA for 96 h, and control cells were treated with ethanol. Cell growth relative to that of the control was plotted against the concentrations of BPA using sigmoid curve fitting and IC_50_ was determined. Here, BPA induced a dose-dependent inhibition of growth in CC-2 cells and 0.1 μM was closer to IC_50_.

To investigated the biological function of BPA in GC-2 cells, we treated the cells with multiple doses of BPA for 96 h, ranging from 1 nM to 1 μM. It showed that BPA inhibited GC-2 cell growth and this effect was dose-dependent (Figure 2). The half-maximal inhibitory concentration (IC_50_) of BPA was almost 0.1 μM. We considered that low dose of BPA could inhibit GC-2 cell growth.

### Activate phosphorylation of Erk1/2 by BPA in mouse GC-2 cells

**Figure 3:**
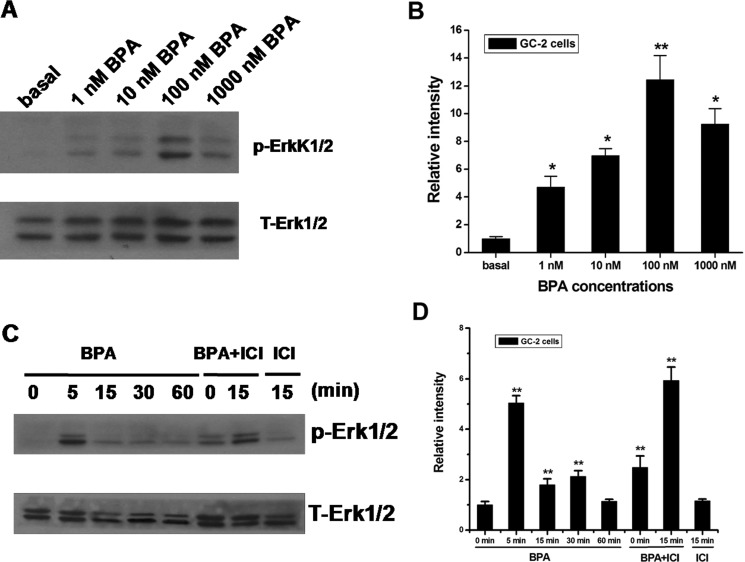
Effects of BPA on Erk1/2 activation in GC-2 cells (**A**, **B**) BPA-induced activation of Erk1/2. Cells were treated for 30 min with the indicated concentrations of BPA, and the phosphorylation of Erk1/2 were examined by western blot. (**C**, **D**) Cells were treated for the indicated times with 100 nM of BPA. For the treatment with an estrogen antagonist, cells were pretreated with 10 μM ICI for 30 min and then treated with 100 nM BPA for 5 or 15 min. Western blot analyses of the amounts of phospho-Erk1/2 (p-Erk1/2) and total Erk1/2 (T-Erk1/2) were performed on 50 μg of total proteins extracted from GC-2 cells untreated (basal) or treated as indicated. Blots are representative of three independent experiments with similar results. **P < 0.01.

Estrogens and xenoestrogens were shown to generate a rapid signal via second messengers, such as Ca^2+^, cAMP and G-proteins, which in turn activate various downstream kinases [47]. To evaluate whether BPA is involved in the rapid cellular response and exerts the estrogenic activity, we investigated the effect of BPA in mouse GC-2 cells on p42 ⁄ 44 MAPK (Erk1⁄ 2) phosphorylation. Using increasing doses of BPA(1 nM −1000 nM) for 30 min, we observed that BPA was able to induce Erk1/2 phosphorylation in GC-2 cells, with 100 nM being the most effective concentration (Figure 3A, 3B).

To investigate the receptor-mediated pathway, GC-2 cells were treated for 30 min with 10 μM ICI 182,780 (ICI), the classical ER antagonist. We investigated the time course of Erk1/2 activation by BPA (100 nM) and found that induction occurs rapidly with a peak at 5 min (Figure 3C, 3D).

It supported that BPA stimulates the phosphorylation mediated by non-classical receptors and this effect is dose- and time- dependent.

### BPA-induced Erk1/2 activation is mediated by GPR30 through EGFR-MAPK pathway in GC-2 cells

**Figure 4:**
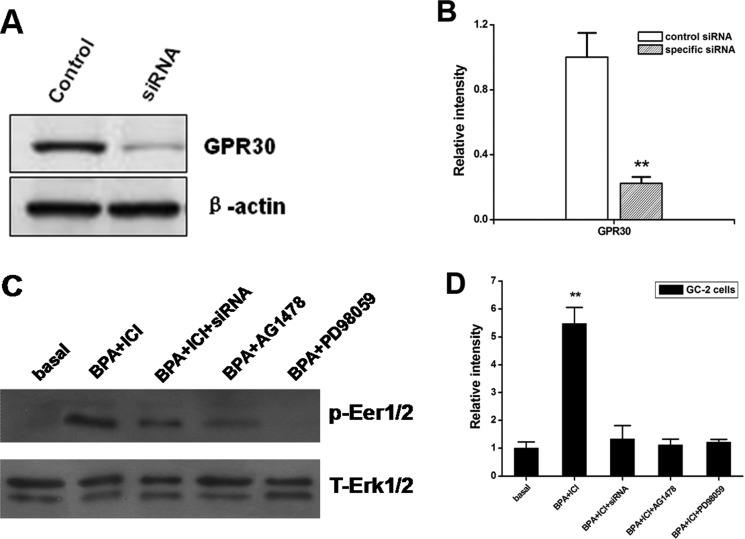
The expression of activated Erk1/2 in GC-2 cells performed by different ways (**A**, **B**) Expression of GPR30 in GC-2 cells transfected with specific siRNA. GC-2 cells were transfected with 100 nM siRNA against GPR30, non-targeting (control siRNA) siRNA as indicated. At 48 h post transfection, protein was extracted and subjected to a Western blot analysis for GPR30. The levels of b-actin protein were used as loading control. Results are representative of three independent experiments. (**C**, **D**) Blocking of Erk1/2 signaling with PD, AG and siRNA against *Gpr30*. Cells were pretreated with 10 μM of ICI, or10 μM PD+ICI or 10 μM AG+ICI for 30 min or *Gpr30* siRNA for 48 h, and then treated with 0.1 μM BPA for 15 min. The activation of Erk1/2 was examined as shown in Figure 4. D. The figure of Western blot is a representative of three independent experiments. The density of the band for p-Erk1/2 was normalized with that of total Erk1/2 and the value is shown in the graph (right). **P < 0.01, compared to the control.

To further characterize the role and function of the novel estrogen receptor GPR30, in the BPA-induced activation of Erk1/2, we carried out small interfering RNA (siRNA) experiments. The expression of GPR30 protein was reduced in GC-2 cells transfected with *Gpr30* siRNA (Figure 4A and 4B). When the expression of *Gpr30* was reduced with specific siRNA, the BPA-induced activation of Erk1/2 was completely abolished (Figure 4C and 4D). Furthermore, BPA-induced activation was completely abolished in the presence of 10 μM MEK inhibitor PD98059 (PD) or 10 μM EGFR inhibitor AG1478 (AG). Therefore, the results suggested that the expression of GPR30 is necessary for the BPA-induced activation of Erk1/2. EGFR-MAPK pathway is involved this process.

### BPA altered the expression level of *c-Fos* mRNA in GC-2 cells

**Figure 5:**
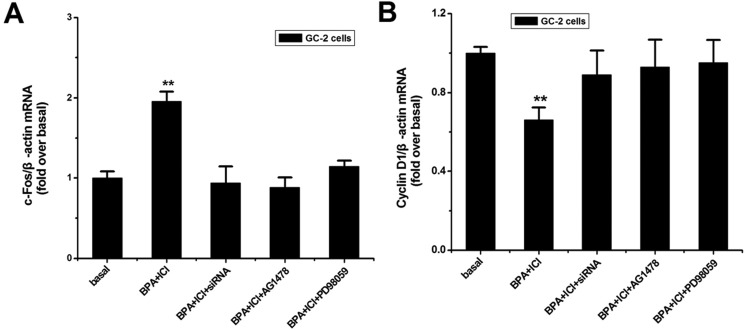
Effects of BPA on the downstream relative genes expression (**A**, **B**) GC-2 cells were treated as described above, Total RNA was extracted for qPCR. *c-Fos a*nd *Cyclin D1* mRNA expression levels were evaluated following normalization to *β-actin* level. The values represent the mean ± SD of the data from three independent experiments. **P < 0.01.

*c-Fos* gene participates in the regulation of cell cycle [48]. And *Cyclin D1* gene is an essential cell cycle regulatory molecule. Therefore, we evaluated the mRNA expression levels of these targets using qPCR and chose *β-actin* as the control. The results in Figure 5 showed that the expression of *c-Fos* was up-regulated and *Cyclin D1* gene was down-regulated, in the presence of BPA and ICI. And in other cases the expression levels of these two genes did not change.

### BPA inhibits GC-2 cell growth though EGFR-MAPK pathway mediated by GPR30

**Figure 6:**
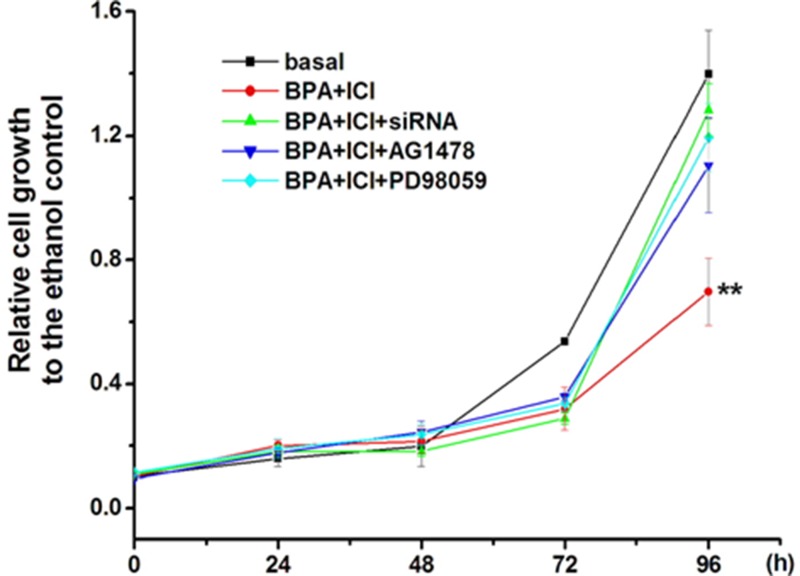
Inhibition of GC-2 cell proliferation by BPA went though EGFR-MAPK pathway mediated by GPR30 GC-2 cells were planted in 96-well plates with a density of 2,000 per well. Incubated with 0.1 μM BPA and examined the cell activity every 24 h using MTT assay. After 72 h treatment, the account of cells number was less compare to the basal, and 96 h later, this number disparity was more apparent (*P* < 0.01). In conclusion, the activation of GPR30 was closer to the cell growth inhibition. siRNA, AG and PD prevented from the inhibition. **P < 0.01.

To confirm the hypothesis that whether BPA inhibited GC-2 cell growth though EGFR-MAPK pathway mediated by GPR30, MTT assay was performed. We monitored the absorbance of formazan which indirectly reflected cell activity and cell population. BPA slowed down the cell growth after 48 h. The activation of GPR30 induced by BPA inhibited the cell growth and ICI. GPR30 siRNA, EGFR inhibitor (AG) and MAPK (PD) inhibitor could partially reverse the effect (Figure 6).

### BPA induce GC-2 cells apoptosis though EGFR-MAPK pathway mediated by GPR30

**Figure 7:**
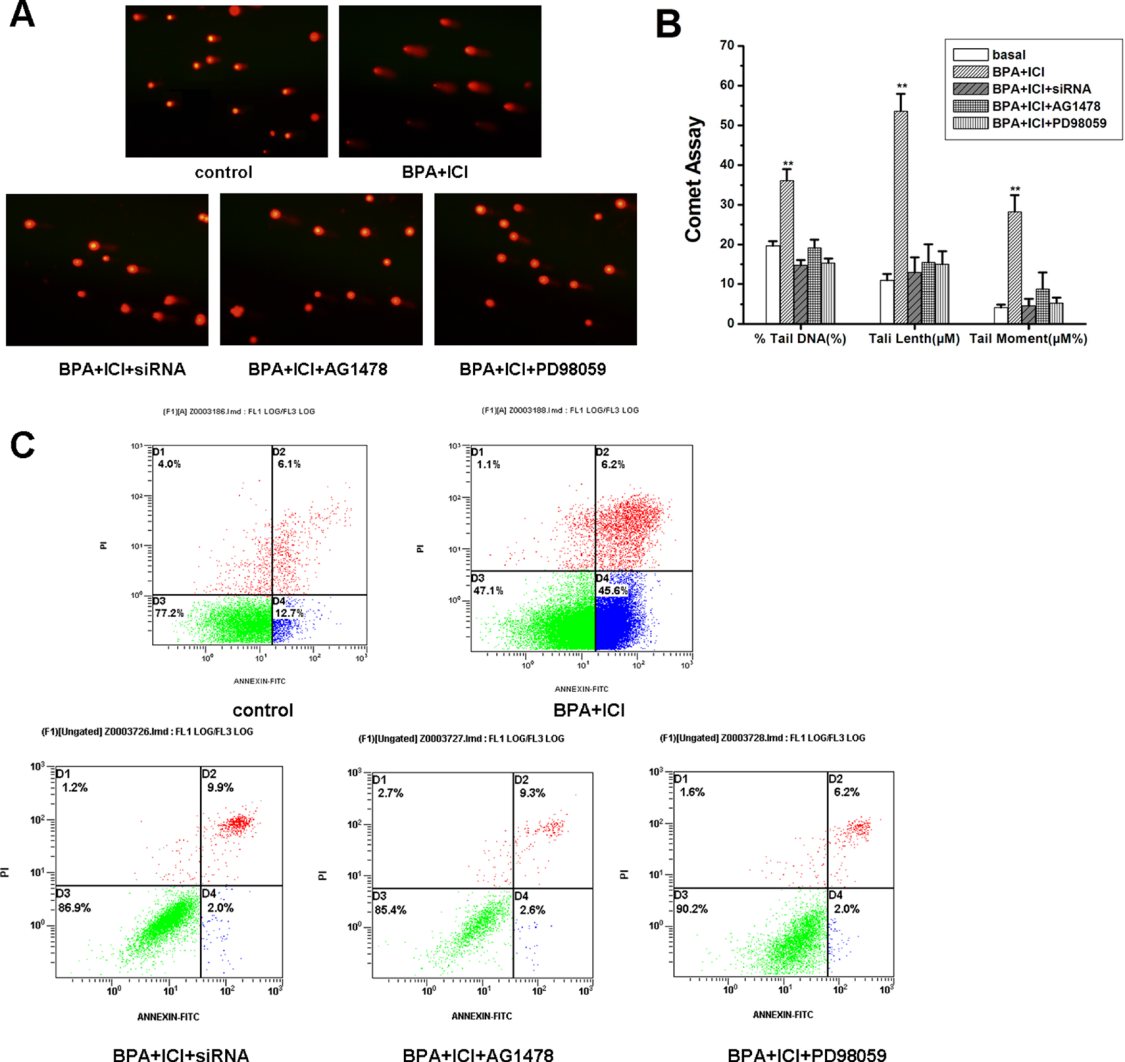
(**A**) Comet assay. DNA-damaged induced in different ways as described above in GC-2 cells. (**B**) The histogram of relative index in Comet assay. (**C**) Flow cytometry. Apoptosis induced in different ways as described above in GC-2 cells.

BPA could inhibit GC-2 cells proliferation, however, the reason is unclear. Comet assay could intuitively show the damaged DNA, and flow cytometry could determine the apoptosis cells. The cells were treated as described above. Damaged- DNA was examined by comet assay (Figure 7A and 7B) and apoptosis cells were examined by flow cytometry (Figure 7C). In these results, we inferred that BPA could lead to GC-2 cells apoptosis, and the process was though the EGFR-MAPK pathway, mediated by GPR30.

### BPA caused mice spermatocyte apoptosis

**Figure 8:**
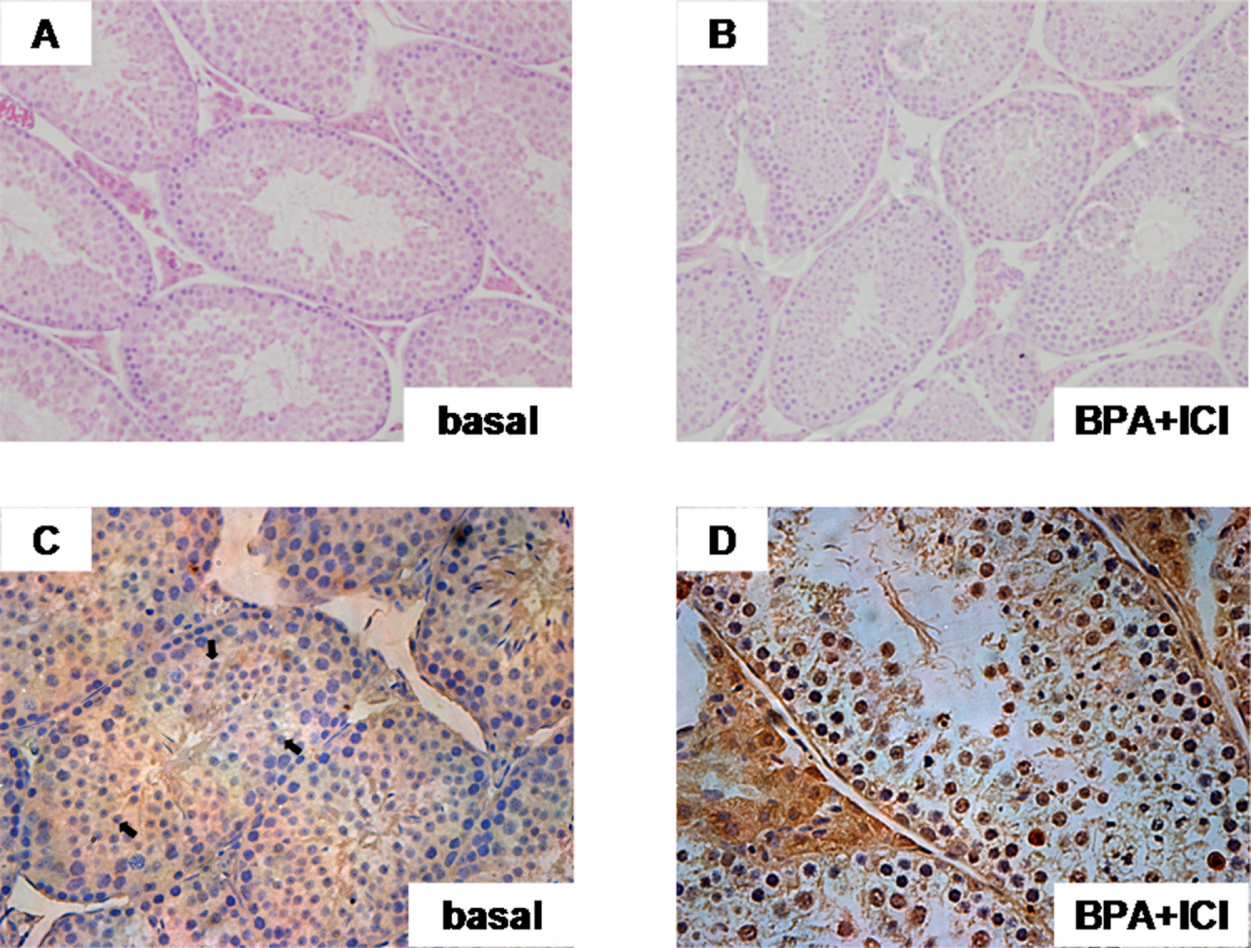
Spermatocytes apoptosis occurred via GRP30-mediated (TUNEL) (**A**, **B**) Seminiferous tubules in normal and BPA-treated mouse. (**C**, **D**) The apoptosis examined by TUNEL assay. Apoptosis triggered by BPA via GPR30 in spermatogenic cells from mouse testis. The arrow pointed to spermatocytes.

The previous results did not provide information on the biological effect of BPA *in vivo*. It is necessary to performed on the live animals. The BPA–damaged model in mice was built. Immunohistochemistry on mice testis indicates that BPA induce spermatocyte apoptosis significantly, without affecting the seminiferous tubules and spermatocyte (Figure 8). Thus, it prompts that BPA, one of EDCs, could harm reproductive system.

## DISCUSSION

BPA, one of the EDCs, has a great impact on human's endocrine system today. Low concentration of BPA is involved in mediating cell proliferation of pancreatic islet, endothelium, breast, pituitary gland, and prostate tissues [15, 47, 49]. BPA acts as xenoestrogen, in which the ability to bind ERs is approximately 1000–10,000 times less than that of natural estrogens [50, 51]. It has been well known that estrogens disrupt human testicular function, mediated by ERs [24–26]. BPA has been usually reported to induce the human reproductive system disorders [52–55].

GPR30 is a novel membrane ER [42] with high-affinity and low-capacity binding to estrogens [43]. In present studies, GC-2 cells expressed ERa and ERb as well as GPR30. It is difficult to make sure which of the three receptors is responsible for the BPA biological action. To eliminate the interruption of ERs, we used selective ERs down-regulator ICI to inhibit the contaction of BPA and ERs. It turned out that BPA should bind to GPR30 to activate the rapid phosphorylation of Erk. GPR30 can mediate the immediate activation of Erk through EGFR [56]. As shown in Figure 3C, our data indicated that the phosphorylation of Erk was activated through EGFR-MAPK pathway mediated by GPR30, which were validated by siRNA and a series of EGFR-MAPK inhibitors.

Furthermore, as the similar methods treated, we investigated the relative expression levels of downstream genes *c-Fos* and *Cyclin D1*. *c-Fos* gene is a proto-oncogene upregulated by numerous stimuli that enhances its expression and interacts with c-JUN to form heterodimers that regulate cell proliferation and differentiation [39, 56]. *Cyclin D1* promotes the G1 to S phase transition in cell cycle, and it plays a specific role in mitosis [57]. In this study, low concentration of BPA increased c-fos gene expression and down-regulated the expression of *Cyclin D1* through EGFR-MAPK pathway. We concluded that BPA inhibits growth of the cells. Then, the comet assay and flow cytometry verified that BPA could induce GC-2 cells apoptosis. Thus, the mechanism of BPA in decreasing GC-2 cells proliferation was proposed as follows: low concentration of BPA could bind to membrane GPR30 and activate the EGFR-MAPK pathway, which caused the activation of *c-Fos* gene and the inhibition of cell-cycle gene *Cyclin D1*.

We also implemented animal experiment to confirm this speculation. The results proved that BPA triggered spermatocyte apoptosis via GPR30, while it didn't alter the morphological structure of mouse seminiferous tubules.

## MATERIALS AND METHODS

### Cell cultures and treatments

GC-2 cells (a mouse spermatocyte-derived cell line; American Type Culture Collection, Manassas, VA) were cultured in DMEM growth medium supplemented with 10% fetal bovine serum (FBS), 1% glutamine, and 1% penicillin/streptomycin (pen/strep) (Invitrogen, USA). Cells were maintained in growth medium for 48 h and then starved for 24 h using DMEM medium supplemented with 1% pen/strep before being treated. Cells were cultured in 60-mm dishes (1 × 10^6^ cells) for RNA and protein extraction, and in 12-well plates for cell proliferation (2 × 10^5^ cells) assay. Treatments were performed at different times using: ICI 182780 (Sigma, USA), siRNA (Qiagen, Germany), PD98059 (Promega, USA), AG1478 (Sigma, USA), Bisphenol A (Sigma, USA).

### RNA extraction, cDNA synthesis and PCR reaction

#### Total RNA extraction

Total RNA was extracted from cell cultures using the Trizol commercial kit (Invitrogen, USA) according to the manufacturer's protocol. Possible genomic DNA contamination was removed from the total RNA preparation using DNA-free^TM^ Kit (Applied Biosystems, Ambion, AM1906). The final content of RNA was assayed with NanoDrop Spectrophotometer ND-1000 (Saveen Werner, Limhamn, Sweden), and the quality was checked by electrophoresis through agarose 1% gels stained with ethidium bromide. Only samples that were not degraded and showed clear 18S and 28S bands under ultraviolet light were used for RT-PCR.

### cDNA synthesis

Intact RNA was converted to cDNA by reverse transcription using TaqMan Reverse Transcription Regents (Applied Biosystems, Foster City, CA, USA), according to protocols from the manufacturer. The final concentration of cDNA was 10 ng/ml verified by NanoDrop Spectrophotometer ND-1000. The samples were stored at −20°C until further used.

### Real-time PCR reaction

The expression of selected genes was quantified by real-time PCR using ABI PRISM 7500 sequence detection system (Applied Biosystems, USA) following the manufacturer's instructions. PCR was performed on 200 ng cDNA using specially primers. Gene-specific primers were designed using Primer premier 5.0 software and primer sequences are listed as follows: c-Fos (forward, 5′-GAGGAGGGAGCTGACAGATACACT-3′; reverse, 5′-GATTGGCAATCTCAG TCTGCA A-3′); Cyclin D1 (forward, 5′- TCCGCA AGCATGCACAGA-3′; reverse, 5′- GGTGGGTTGGAAATGAACTTC A-3′). We used β-actin (forward, 5′- CTGGAACGGTGAAGGTGACA-3′; reverse, 5′- AAGGGACTTCCTGTAA-3′) as a reference gene. We performed quantitative real-time PCR using Immolase heat-activated Taq DNA polymerase (Invitrogen, USA). SYBR Green I (Invitrogen, USA) was used for detecting fluorometric product. Cycle parameters were 96°C for 15 min for polymerase activation, followed by 40 cycles of 95°C for 15 sec, 57°C for 15 sec, and 72°C for 30 sec, with an optical read stage at 83.5°C for 6 sec.

### Western blot analysis

Cells were grown in 10-cm dishes after treatment and GC-2 cells were washed in PBS and homogenized in buffer (10 nM Tris-HCl, 5 mM EDTA, 50 nM NaCl, 50 mM sodium fluoride, 30 mM sodium pyrophosphate, 1% Triton-X, 200 μM sodium orthovanadate, 1 mM phenyl methyl sulfonyl fluoride, 1 μg/mL pepstatin, 2 μg/mL leupeptin, 5 μg/mL aprotinin). The protein concentration was determined using the Pierce (Rockford, IL) BCA (bicinchoninic acid) protein assay.

Equal amounts of whole protein extract were electrophoresed onto 12% or 15% sodium dodecyl sulfate polyacryl amide gel electrophoresis (SDS-PAGE) replicas of gels and then transferred to nitrocellulose membranes (Millipore, Billerica, MA) using a semi-dry transfer cell (Bio-Rad Laboratories, Hercules, CA) at 1 mA/cm2 for 2 h. After transferred to polyvinyl difluoride membranes, samples were blocked in 1% dry milk and incubated over-night with the following primary antibodies

ERα (1:400; Santa Cruz Biotechnology, Santa Cruz, CA), ERβ (1:3,000; Upstate, Danvers, MA), GPR30 (1:1000; Santa Cruz Biotechnology, Santa Cruz, CA) or GAPDH (1:10,000; Sigma). After incubation with horseradish peroxidase–conjugated secondary antibody (Amersham, Piscataway, NJ), products were developed on film using Super Signal chemiluminescence reagents (Pierce).

The levels of proteins and phosphoproteins were detected with horseradish peroxidase-linked secondary antibodies and revealed using the ECL^®^ System (GE Healthcare, Milan, Italy).

### MTT assay

Cells were plated at a density of 6,000 or 8,000 cells/ well in 96-well plates in plating medium. For the determination of cell growth, treatments were performed for 48 h in serum-free medium without phenol red. Control cells were treated with the same amount of DMSO (0.1% DMSO) alone, which never exceeded the concentration of 0.01% (vol/vol). The treatment effects on GC-2 cell growth were determined by MTT (3-[4, 5-dimethylhiazol-2-yl]-2, 5-diphenyltetrazolium bromide) assay; In brief, at the end of treatment, 100 μl of MTT (5 mg/ml in 1M PBS, pH 7.6) was added to each well of 96-well plates and allowed to incubate for 2 h at 37°C in 5% CO_2_ air. The optical density (OD) was measured using a microplate reader at 570 nm. MTT was added at a final concentration of 0.5 mg/mL for 2 hr. After medium aspiration, the formazan dye was extracted with DMSO and absorbance was read at 570 nm using a plate reader (Bio-Tek, Winooski, VT).

### Single cell gel electrophoresis (comet assay)

1 × 10^6^ cells were suspended in 1 ml PBS (137 mM, 2.7 mM KCl, 10 mM Na2HPO4, 2 mM KH2PO4, PH 7.4). 10 μl suspensions was mixed with 90 μl 1% low melting agarose and placed onto a slide pre-coated with 1% low melting agarose. Then the suspension was immediately covered with a clean coverslide and kept for 10 minutes in order to solidification. The coverslide was slightly removed and 500 μl lysis buffer (2.5 M NaCl, 100 mM Na2-EDTA, 10mM Tris, PH 10, 1% Triton X-100) was dropped on the embedded cells for 1.5 hours. After washing in re-stilled water, the slide was placed in a horizontal gel electrophoresis chamber filled with cold electrophoretic buffer (0.3 mM NaOH, 1mM Na2-EDTA, PH 13) for 25 minutes to make the DNA unwind, and then performed electrophoresis for 25 minutes (25V, 300 mA). After that, the slide was treated with re-stilled water one time and washed with the residual electrophoretic buffer and neutralization buffer (0.4 M Tris, PH 7.5) 3 times. All the steps were performed in dark and cold condition to prevent additional DNA damage. The slide was stained with ethidium bromide (20 mg/ml), and then analyzed with a fluorescence microscope (Leica DM IRB) at a magnification of 200 ×. Digital images were acquired with DP-BSW software (OLYMPUS) and analyzed by the CASP software. More than 100 cells were analyzed for each sample.

### Annexin V/propidium lodide assay for apoptosis detection

Before cells were collected by centrifugation and resuspended in 500 μl annexin-binding buffer. The concentration of final cells suspension was approximate 1 × 10^6^ cells, which was determined on a hemocytometer. Each sample was added 5 μl of Annexin V-FITC and 10 μl of propidium iodide and incubated on ice in the dark for 5 minutes, and then analyzed on a flow cytometer (BD Biocsiences, San Jose, USA). To discriminate between positive and negative group, a non-stained control sample was subjected to flow cytometer acquisition and analyzed to define their cut off. The collected fluorescence for annexin V was at 530/30 nm and for PI was at 584/40 nm. Data were analyzed with BD FACSuite ^TM^. Data were collected on more than 10,000 cells per cell sample.

### RNA interference

GPR30 siRNA and non targeting siRNA were purchased from Qiagen. GC-2 cells were plated into 60 mm dishes at 1 × 10^6^ cells for protein extraction, and into 24-well plates at 2 × 10^5^ cells/well for proliferation assay and used for transfection 24 h later. The cells were transfected using the siRNA transfection reagent (Qiagen, Biotechnology) with 10 nM GPR30 or control siRNA according to the manufacturer's instructions. The oligonucleotides used were: 5′-TGGAGTAGTCGCATCCAT-3′ for GPR30 and 5′-GATCTCAGC ACGGCAAAT-3′ for the scrambled control. Immunoblotting and the quantitative real-time reverse transcription-polymerase chain reaction (qPCR) were then performed. To confirm the specific inhibitory activity, we carried out a Western blot analysis using the antibody against GPR30. For inhibited proliferation experiments, cells were maintained in medium containing the transfection mix for 24 h and medium was then replaced for the treatment. Proliferation was evaluated 48 h later.

### Immunohistochemistry

Serial paraffin wax sections of mice testis (20 μm) were analyzed using TUNEL assay (Boster, Wuhan, China). Briefly, the sections were incubated with labeling buffer containing TdT and DIG-d-UTP for 120 min at 37°C to incorporate adequately and were then turned to incubate with diluted extraavidin-peroxidase link for another 30 min at 37°C, followed by the addition of DAB at room temperature for 30 min. All sections were counterstained with hematoxylin. We scored the cells of which the expression developed a brown color as positive ones.

### Statistical analysis

Statistical differences were determined by one-way analysis of variance followed by Newman-Keuls post hoc analysis. *P*-Values < 0.05 were considered significant. All experiments were performed at least three times.
